# The Absorption of Needle-Free Insulin Aspart Through Jet Injector in Different Body Parts of Healthy Individuals

**DOI:** 10.3389/fendo.2022.832726

**Published:** 2022-04-29

**Authors:** Qi Pan, Lina Zhang, Aimin Gu, Dongni Yu, Xiaoxia Wang, Yan Zhou, Lixin Guo

**Affiliations:** Department of Endocrinology, Beijing Hospital, National Center of Gerontology, Beijing, China

**Keywords:** needleless syringe, hyperinsulin-normal glucose clamp, injection site, insulin absorption, healthy adult male

## Abstract

The absorption of needle-free fast-acting insulin injected into different body parts of healthy male subjects was studied in an attempt to provide clinical guidance for diabetic patients who take needle-free insulin injections in terms of providing reference in the clinical guidance regarding the correct use of needle-free insulin injections among diabetic patients. This randomized, open-label, cross-over trial was conducted on eight healthy adult male volunteers, in which the skin thickness at three injection sites (abdomen, upper arm, and thigh), the time to peak, peak rate, and area under the glucose infusion rate (GIR) curve of plasma insulin were measured through the hyperinsulin-normal glucose clamp test after the injection of insulin aspart with a needle-free syringe at three different sites to analyze the correlation between insulin absorption index at different injection sites and skin thickness. The values of the skin thickness of the abdomen, upper arm, and thigh measured by ultrasonic wave were 2.45 ± 0.34 mm, 2.18 ± 0.50 mm, and 1.93 ± 0.55 mm, respectively. There was a significant difference in the skin thickness of the abdomen and thigh (P = 0.014). The hyperinsulin-normal glucose clamp model was successfully established for each subject. Approximately 0–2 h after injection of insulin aspart with needle-free syringes, the area under the GIR-time curve of the abdomen, upper arm, and thigh was 29,400.75 ± 2,645.00 ml, 30,230.50 ± 4,937.87 ml, and 30,179.63 ± 6,188.57 ml, respectively. There was no significant difference in the area under the GIR curve between any two injection sites (P >0.05). The time to peak of GIR at different injection sites was 38.68 ± 13.57 min in the abdomen, 40.86 ± 12.70 min in the upper arm, and 37.03 ± 13.29 min in the thigh, respectively, in which no significant difference was found between each of them (P >0.05). The GIR curve after injection at the three different sites was consistent with each other. There was no significant difference in insulin absorption after the injection of insulin aspartate into the abdomen, upper arm, and thigh with a needleless syringe in healthy male adult volunteers, and there was no correlation between skin thickness at the injection site and insulin absorption. Injection sites did not affect the absorption of insulin in needle-free injections.

## 1 Introduction

Diabetes mellitus (DM) is a clinical syndrome characterized by chronic hyperglycemia due to inadequate insulin secretion and/or deficient insulin action. Chronic complications of diabetes can occur because of prolonged poor glycemic control (1). Good glycemic control can improve the progression of diabetes-related complications.

Diabetes is a progressive disease. In type 1 diabetes, insulin must be used to maintain life, while in many patients with type 2 diabetes, it is necessary to increase the drug dose or choose combination drug therapy for better glycemic control (2). When the combined use of oral hypoglycemic agents is not effective or conjunctive, insulin or non-insulin injections should be used to control hyperglycemia and reduce the risk of diabetic complications. For patients with a long course of disease or multiple complications, insulin therapy has become the most important and even necessary measure to control blood glucose.

Insulin administration technology has undergone a long process of continuous innovation (3). The advent of insulin pens has improved insulin delivery. Compared with syringes, it is simpler, safe, effective, operable, and easy to carry and improves the treatment compliance of a patient to a certain extent. With the development of injection technology, needleless syringes have been accepted by a large number of patients due to their advantages, such as good injection experience, less subcutaneous injury, and faster peak time of efficacy, thus becoming a new choice for diabetic patients with insulin injection.

Needle-free injectors, called jet injectors, are usually used for insulin administration among patients who have injection-related anxiety or phobia. Additionally, the use of these jet injectors has been reported to markedly improve and accelerate the absorption of rapid-acting insulin from subcutaneous tissues and into the blood (4). These injectors often deliver insulin at a very high velocity, reaching more than 100 m/s across the subcutaneous tissue while distributing insulin over a larger area compared to syringes (5). Recent evidence shows that the use of the euglycemic clamp technique is effective among healthy individuals in terms of shortening the time-to-peak plasma insulin levels by as much as 50% compared to the normal insulin injection pen (6).

When patients start to use insulin injection therapy, it is necessary to choose the injection site for each injection. Current studies have shown that when insulin is injected into patients through a syringe, injection sites may impact on the insulin absorption and blood glucose control of patients (7). However, there are still no relevant studies on needle-free insulin injections. In this study, the normal glucose clamp technique was used to observe the effects of the injection at different injection sites in the body with needle-free syringes on the absorption of insulin aspart.

## 2 Materials and Methods

### 2.1 Research Subjects

In this randomized, open-label, cross-over trial, we included healthy male subjects (18–75 years of age) at the Beijing Hospital, Beijing, China, during March, 2021. The inclusion criteria included the following: (1) healthy male subjects with no diabetes or any other endocrine abnormalities, (2) clinical examination and laboratory assessment revealing no abnormalities, (3) body mass index (BMI) ranging from 18 to 32 kg/m^2^ with no change in body weight of more than 10 kg in the 3 months preceding screening, and (4) hemoglobin ≥12 g/L during screening.

On the other hand, patients were excluded if they had any of the following criteria: (1) participation in other clinical trials within the 3 months preceding the conduct of this trial, (2) severe recurrent hypoglycemia, (3) history of complications related to poor glycemic control such as diabetic ketoacidosis or hyperosmolar coma in the previous 6 months, (4) serious cardiovascular events within the previous 6 months, (5) immunocompromised state, (6) end-stage renal disease requiring dialysis, (7) history of cancer in the previous 5 years, (8) mental disorders, (9) long-term alcohol and drug abuse, (10) chronic skin lesions at the injection sites, (11) allergy to insulin or its preparations, and (12) hemoglobin disorders such as thalassemia or sickle cell anemia or anemia of any cause.

### 2.2 Trial Ethics

This clinical trial was carried out in Beijing Hospital and was approved by the Drug Clinical Trial Ethics Committee of Beijing Hospital (Approval Number: 2020BJYYEc-026-01) (ChiCTR2100049569). This study was conducted in accordance with the Helsinki Declaration, Quality Management Practice for Pharmaceutical Clinical Trials (GCP) and relevant regulations. The subjects were informed of the nature, purpose, and possible adverse reactions before being enrolled in the trial, and all participants gave informed consent before conducting the study protocol.

### 2.3 Research Methods

The establishment of the hyperinsulin-normal glucose clamp model was carried out in four main steps. The first step is the establishment of venous blood collection and infusion channels. Subjects were placed in the supine position, and then a superficial venipentesis was performed on one side of the forearm in the opposite direction of the heart, and an indwelling catheter was placed to maintain the channel with heparin saline to facilitate blood collection. Arterial blood was arterialized by heating the arm of the subject with a constant temperature electric blanket (50–60°C). A venous cannula was placed from the midcubital vein on the other side for insulin and glucose infusion. The second step involved injecting of short-acting human insulin at the beginning of the experiment into an injection pump through the mid-cubitus vein. The infusion rate for the first 10 min was 2.0 mU/kg/min, which rapidly increased the blood insulin level; subsequently, continuous infusion was carried out at a rate of 1.0 mU/kg/min for 290 min. During the period, 0.1 ml of venous blood was taken from the superficial venous channel of the forearm of the subjects to measure the blood glucose value as follows: Blood samples were collected every 5 min during the period of 120–0 min before drug administration and every 5 min during the period from 0 to 60 min (inclusive) after drug administration. Blood was sampled every 10 min during the period from 61 to 180 min (inclusive) after drug administration. The third step was the determination of the target blood glucose value: venous blood was collected every 10 min to measure blood glucose with the glucose oxidase method with the rapid blood glucose detector of Johnson & Johnson (China) Medical Equipment Co., Ltd.). The mean value of three points was taken as the baseline blood glucose value, and the difference between the mean value of baseline blood glucose and 0.28 mmol/L (5 mg/dl) was taken as the target blood glucose value of the subjects in this clamp test. The fourth and final step involved the determination of glucose infusion rate (GIR), where, in the test, the input rate of 20% glucose solution in an infusion pump was adjusted according to the results of blood glucose testing to maintain the blood glucose level at the target value. The GIR and corresponding times were recorded.

#### 2.3.1 The Overall Research Steps

**Figure 1:**
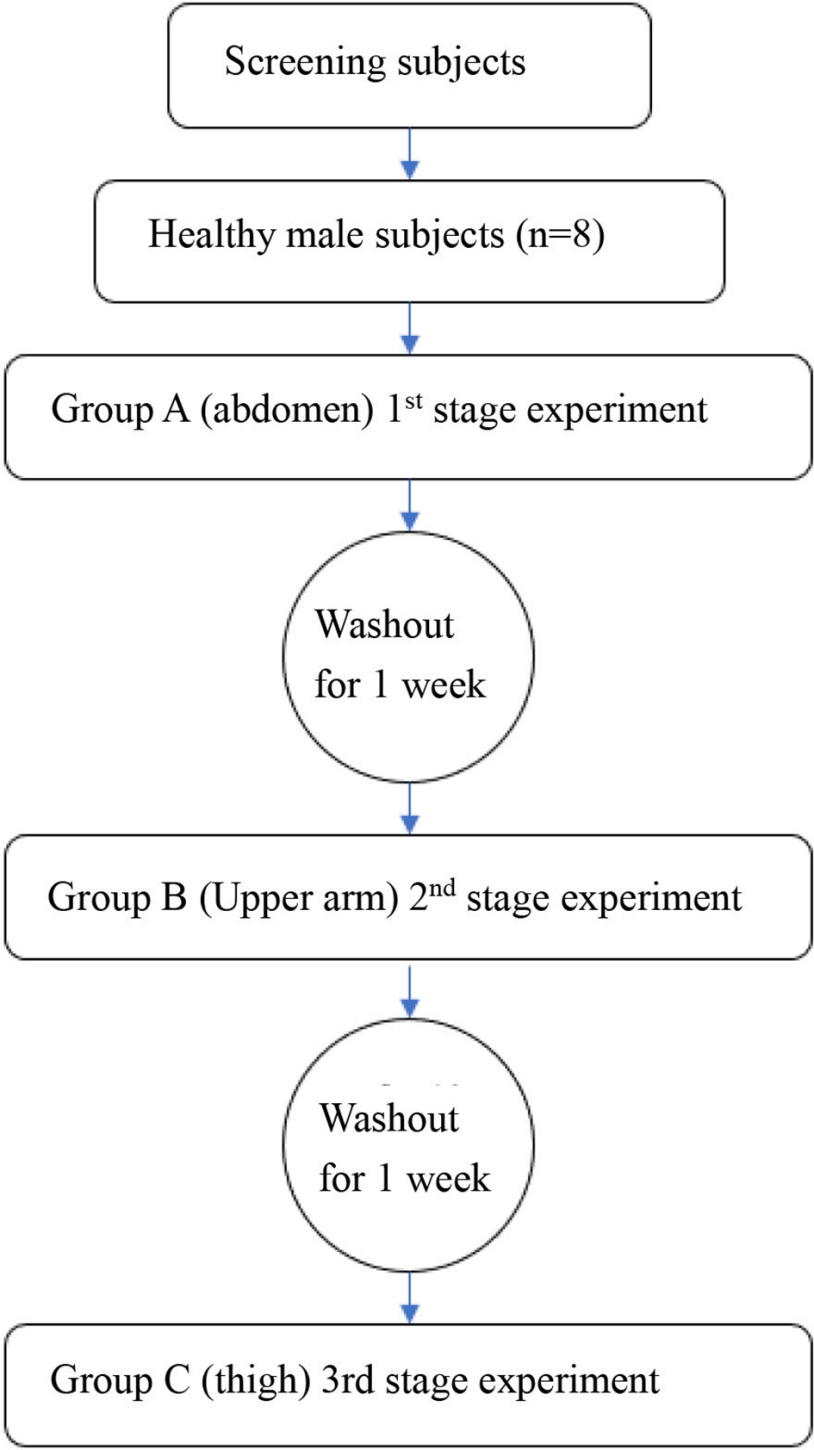
The overall research step. The machines and methods for measuring skin were supplemented. A Samsung RS-80A ultrasonic diagnostic instrument and a L3-12AMHz linear array probe were applied for exploration. All subjects took the supine position, and the skin thickness was measured on the upper abdomen, lateral upper arm, and lateral thigh.

The participation time of each subject was defined as the period from the time when the participants agreed to enter the group to participate in the study to the end of the final follow-up, including screening period of 1 week, the first phase of 1 week, the second phase of 1 week, the third stage of 1 week and the follow-up period of 1 week, so each participant is expected to participate in the study for 5 weeks (the overall flow chart is shown in (**Figure 1**). The skin thickness of the abdomen, upper arm, and lateral thigh in all subjects was measured by ultrasound and recorded. All the subjects fasted for 12 h before the study and then started the phase I trial (start time was recorded): Under the condition of maintaining the same temperature, humidity, environment, and other external factors, all subjects began the study at the same time. After the model of normal glucose clamp was established, all the subjects were injected with insulin aspart (NovoRapid. Penfill, Novo Nordisk. AS) through the QS-M needle-free jet injector (Beijing QS Medical Technology Co., Ltd., China), which was approved by China Food and Drug Administration (FDA) in 2012. The dose was calculated at 0.2 IU/kg. Meanwhile, blood glucose was continuously monitored for 3 h, exogenous glucose infusion rate was adjusted, and GIR changes were recorded. Blood glucose was measured every 5 min 1 h before drug administration and every 10 min 2 h after drug administration. The washout period started at the end of the first phase of the experiment. It was ensured that the subjects kept a normal light diet for a week (weight change within ±5%). The second stage test was started after blood glucose went normally (the start time was recorded): the normal glucose clamp model was reestablished according to the method in the first cycle. The injection experiment was conducted after the successful establishment of the model. The experimental method and the blood sampling time were the same as before, except the injection site was changed into the upper arm. Furthermore, the results were recorded. The washout period started at the end of the first phase of the experiment. It was ensured that the subjects kept a normal light diet for a week. The third stage test was started after blood glucose had gone normally (the start time was recorded): the normal glucose clamp model was reestablished according to the method in the first cycle, and the injection experiment was conducted after the successful establishment of the model. The experimental method and the blood sampling time were the same as before, except the injection site was moved to the lateral thigh. Furthermore, the results were recorded. At the end of each experimental period, the healthy subjects were required to remain under observation for 4 h. Before they left the test center, their vital signs were detected, blood glucose levels were monitored, and adverse events were closely observed and recorded. They can leave with no abnormalities. Follow-up was conducted 7 ± 3 days after the end of the third cycle experiment.

### 2.4 Statistical Analysis

Statistical analysis was performed by SAS 9.4 software (SAS Software Institute in North Carolina, USA). Unless otherwise specified here, all the statistical analysis tests adopted two-sided hypothesis tests, and the level of hypothesis test was set at α = 0.05. That is, P-values less than or equal to 0.05 were considered statistically significant. In normally distributed data, the means and standard deviations (SD) were reported for continuous variables, and the numbers and percentages were reported for categorical variables. On the other hand, for skewed data, the median and interquartile ranges (IQR) were reported for continuous variables. For grade indicators, such as various scoring values, frequency tables were listed, the median and quartile spacing were used for the statistical description, and the rank sum test was performed. For the counting index, frequency tables and percentages were used for description. Chi-square or exact probability methods were used for the test.

The difference in skin thickness at the injection site, the difference in time to peak glucose infusion rate at each site, the difference in peak value of glucose infusion rate at each site, and the difference in area under the GIR curve at each injection site are all compared pairwise by paired T-test. P <0.05/2 = 0.017 (the test level of 0.05 needs to be corrected to reduce class I errors)) means that the difference between the two positions is statistically significant. P >0.05/2 = 0.017 (the test level of 0.05 needs to be corrected to reduce class I errors) means that the difference between the two positions is not statistically significant.

A correlation test was conducted between the thickness of the injection site and the time to peak and the peak rate of plasma insulin. The area under the GIR curve, P <0.05 indicates that the correlation is statistically significant, and P >0.05 means that there is no statistical significance in the correlation. The maximum value of the absolute value of the correlation coefficient R is 1, and the minimum value is 0. The closer to 1, the greater the correlation is. The positive number of the correlation coefficient indicates that there is a positive correlation, and the negative number indicates that there is a negative correlation.

## 3 Results

### 3.1 Demographic Characteristics of the Subjects

Eight subjects were finally included in the analysis, with an average age ranging from 18 to 33 (average = 27.9; mean = 27.47; SD = 13.51) years, a median height of 174 cm (174.00 ± 0.44), and a mean BMI of 22.42 ± 5.15 kg/m^2^. All subjects had no previous family history of diabetes, liver or kidney diseases. All of the participants were not sensitive to the injected insulin aspart, and all of them had a normal hemoglobin level of ≥12 g/L. The baseline demographic characteristics of the included subjects are shown in **Table 1**.

**Table 1:** Demographic characteristics of the subjects.

	Age (Years old)	Body Height (cm)	Body Weight (kg)	BMI (kg/m^2^)
N (Missing)	8 (0)	8 (0)	8 (0)	8 (0)
Mean ± SD	27.47 ± 13.51	174.00 ± 0.44	67.99 ± 8.74	22.42 ± 5.15
Median	27.90	174.00	67.50	22.01
Min, Max	19.97, 32.74	161.00, 183.00	53.00, 82.00	19.07, 26.17

SD, Standard deviation; N, Number.

### 3.2 Difference in Skin Thickness of the Injection Site of the Subjects

All subjects fasted for 12 h before the study. The skin thickness of the abdomen (about 1 cm above the pubic symphysis, higher than the part about 1 cm below the costal margin and on the right of the area surpassing 2.5 cm around the periumbilical), the upper arm (the central 1/3 in the lateral part of the right upper arm) and the lateral thighs (the upper 1/3 part on the anterolateral right thigh) were measured by ultrasound and recorded, as shown in **Table 2**. The skin thickness of the abdomen, upper arm, and thigh was 2.45 ± 0.34 mm, 2.18 ± 0.50 mm, and 1.93 ± 0.55 mm, respectively. Statistical analysis by paired t-test showed that there was a significant difference in the skin thickness of the abdomen and thigh (P = 0.014).

**Table 2:** Difference in skin thickness of the injection site.

Site	Skin thickness (mm)	Paired T	P-value
Abdomen	2.45 ± 0.34	1.676	0.138
Upper arm	2.18 ± 0.50		
Abdomen	2.45 ± 0.34	3.241	0.014
Thigh	1.93 ± 0.55		
Upper arm	2.18 ± 0.50	0.977	0.361
Thigh	1.93 ± 0.55		

The results of paired t-test above showed that P <0.05/2 = 0.017 (the test level of 0.05 should be corrected here to reduce class I error) indicated that the difference between the two parts was statistically significant, P >0.05/2 = 0.017 (the test level of 0.05 needs to be corrected here to reduce class I error) indicated that the difference between the two parts is not statistically significant.

### 3.3 Insulin Absorption at Different Injection Sites

**Figure 2:**
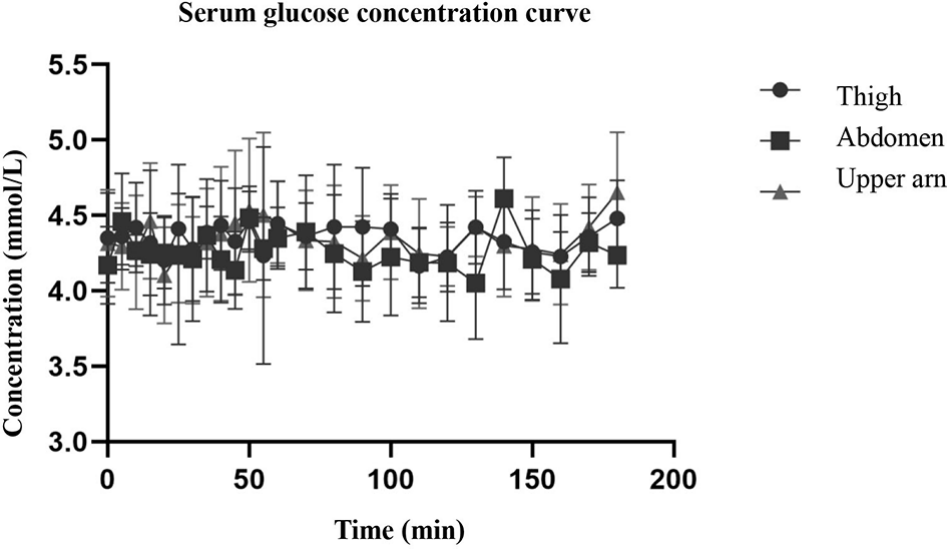
The figure shows that the serum glucose concentrations of the three different injection sites were basically the same as the target blood glucose, and the blood glucose level within 3 h was basically maintained at the target blood glucose level.

**Figure 3:**
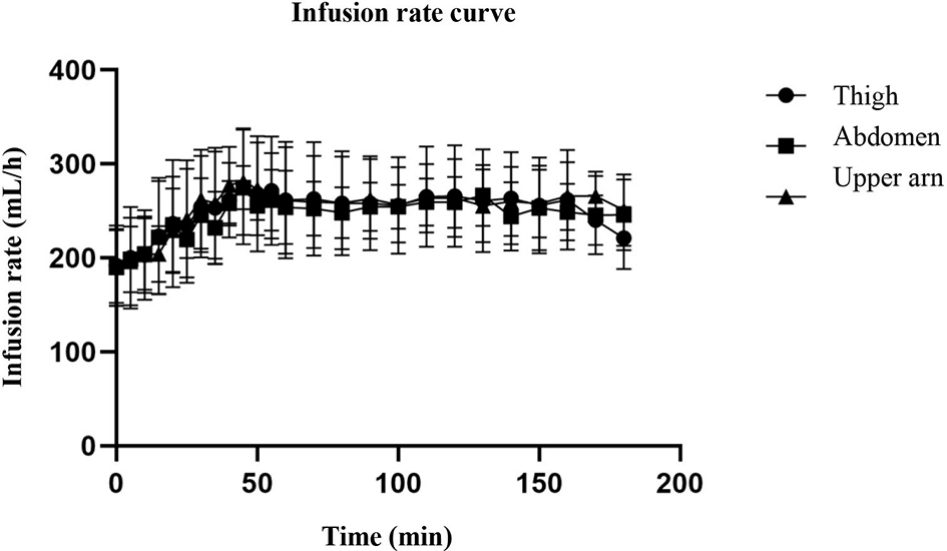
The curve of glucose infusion rate. The figure shows that the serum glucose concentrations of the three different injection sites were basically the same as the target blood glucose, and the blood glucose level within 3 h was basically maintained at the target blood glucose level. As can be seen from the figure, the glucose infusion rate curves of the abdomen, upper arm, and thigh were basically the same after injection.

The hyperinsulin-normal glucose clamp technique was used in this study. During the test, the infusion rate of 20% glucose solution was adjusted according to the blood glucose test results to maintain the target blood glucose level. **Figure 2** shows that the serum glucose concentrations of the three different injection sites were basically the same as the target blood glucose, and the blood glucose level within 3 h was basically maintained at the target blood glucose level, indicating that the normal glucose clamp model was successfully established. Under these conditions, the glucose infusion rate reflected the sensitivity of the tissue to exogenous insulin. As can be seen from **Figure 3**, the glucose infusion rate curves of the abdomen, upper arm, and thigh were basically the same after injection.

The time to peak of glucose infusion rate at different injection sites was 38.68 ± 13.57 min for abdominal injection, 40.86 ± 12.70 min for upper arm injection, and 37.03 ± 13.29 min for thigh injection, as shown in **Table 3**. Paired t-tests showed that P >0.05, indicating that there was no statistically significant difference in the time to peak glucose infusion rate between any two injection sites in the abdomen, upper arm or thigh.

**Table 3:** The differences in time-to-peak, peak values, and area under the glucose infusion rate curve at different injection sites.

Variable	Thigh	Upper Arm	Thigh	Abdomen	Upper Arm	Abdomen
**Time to peak**						
Time	37.03 ± 13.29	40.86 ± 12.70	37.03 ± 13.29	38.68 ± 13.57	40.86 ± 12.70	38.68 ± 13.57
Paired T	-0.584		-0.277		0.344	
P	0.578		0.79		0.741	
**Peak values of GIR**						
Rate (ml/h)	301.38 ± 57.78	310.38 ± 57.39	301.38 ± 57.78	297.75 ± 18.15	310.38 ± 57.39	297.75 ± 18.15
Paired T	−0.568		0.204		0.739	
P	0.588		0.844		0.484	
**Area under the GIR curve (0–2 h)**						
Mean (SD)	30,179.63 ± 6,188.57	30,230.50 ± 4,937.87	30,179.63 ± 6,188.57	29,400.75 ± 2,645.00	30,230.50 ± 4,937.87	29,400.75 ± 2,645.00
Paired T	0.043		−0.539		−0.726	
P	0.967		0.607		0.491	

P, P-value; GIR, Glucose infusion rate. The results of paired t-test above showed that P <0.05/3 = 0.017 (the test level of 0.05 should be corrected here to reduce class I error) indicated that the difference between the two parts was statistically significant, P >0.05/3 = 0.017 (the test level of 0.05 needs to be corrected here to reduce class I error) indicated that the difference between the two parts was not statistically significant.

The peak values of the glucose infusion rate at different injection sites were 297.75 ± 18.15 for abdominal injection, 310.38 ± 57.39 for upper arm injection, and 301.38 ± 57.78 for thigh injection, respectively, as shown in **Table 3**. The paired t-test showed that P >0.05, indicating that there was no statistically significant difference in the peak value of glucose infusion rate between any two injection sites in the abdomen, upper arm, or thigh.

The changes in plasma insulin content at different injection sites are compared in **Table 3**. The results of the paired t-test showed that P >0.05, which meant that there was no statistically significant difference in the area under the glucose infusion rate-time curve between any two injection sites in the abdomen, upper arm, or thigh 0–2 h after insulin injection, indicating that the absorption of insulin at different injection sites was similar.

The correlations between skin thickness at different injection sites, time to peak, and peak rate of plasma insulin, and area under the GIR curve are shown in **Table 4**. The correlation coefficients were all small (R was −0.084, 0.006, and −0.055, respectively), and P-values were all greater than 0.05, indicating that there was no statistically significant correlation between skin thickness at the injection sites and time to peak, peak rate of plasma insulin, and area under the GIR curve.

**Table 4:** Correlation between skin thickness at injection sites, time to peak and peak rate of plasma insulin and the area under GIR curve.

Indices	Skin thickness	
	(r)	P-value
Time to peak	−0.084	0.695
Peak rate	0.006	0.976
**Area under GIR curve**	−0.055	0.800

P <0.05 meant the correlation was statistically significant, P <0.05 indicated no statistical significance. The maximum value of the absolute value of the correlation coefficient R is 1, and the minimum value is 0. The closer to 1, the greater the correlation is. The positive number of correlation coefficient indicates that there is a positive correlation, and the negative number indicates that there is a negative correlation.

## 4 Discussion

For diabetic patients who cannot control the disease with oral hypoglycemic drugs, the supplementation of exogenous insulin is one of the main therapeutic methods for better glycemic control. In the past decades, great progress has been made in insulin therapy, new insulin preparations, and administration methods have been developed, but there are still many obstacles, challenges, and uncertainties (8). The main reasons for patient refusal were inconvenient of insulin treatment (51.6%) and fear of injection (48.2%) (9). Due to the long course of disease, patients receiving insulin injections generally ignore the injection site rotation strategy and often reuse needles, which leads to subcutaneous fat hypertrophy (10–15). This, in turn, affects glycemic control, resulting in unexplained hypoglycemia and a significant increase in blood glucose change levels (11, 16, 17), which leads to an increase in insulin costs (18).

Needleless injection is a new type of insulin administration technology that is simple, safe, effective, operable, and easy to carry. It can reduce the fear of syringes and significantly improve the quality of life of patients (18). Insulin injected by injection shows a specific tapered diffusion pattern in subcutaneous tissues with a relatively large surface area. This diffusion pattern enhances the absorption of insulin in the blood circulation, thus achieving a more direct hypoglycemic effect (10). The injection of insulin aspart by jet can enhance insulin absorption and shorten the duration of hypoglycemic action. This curve is more similar to the pattern of endogenous insulin secretion, which can achieve better postprandial insulin coverage and correct postprandial glucose fluctuations (6).

Previous studies have shown (19) that in patients with type 1 and type 2 diabetes, insulin absorption is significantly faster and postprandial hyperglycemia is significantly reduced after the administration of a needleless jet syringe. The improvement of early postprandial glucose control may be particularly beneficial for patients with difficulty controlling postprandial glucose fluctuations (20). Compared with traditional needle-injected insulin therapy, needle-free insulin therapy showed a better hypoglycemic effect and significantly improved the satisfaction of patients with insulin therapy. Additionally, needle-free syringes also have better safety (21). However, there are few studies on whether needle-free injection is affected by skin thickness at different injection sites.

The hyperinsulin-normal glucose clamp technique was used in this study. The observational record indices under study included: (1) changes in the plasma insulin levels of the subjects; (2) changes in the GIR curve of the subjects; and (3) skin conditions of the subjects at the injection sites (injection reaction, skin thickness). Through the comparison of these indicators, the differences in drug absorption and safety of insulin injected with needle-free syringes in different parts of the body were studied.

Although the skin thickness at the three injection sites was different, especially that of the abdomen and thigh, there was a significant difference (**Table 2**), but the serum glucose concentration curve of the three parts after insulin injection (**Figure 2**) was basically consistent with the glucose infusion rate curve (**Figure 3**). A significant difference was not seen between time and peak (**Table 3**) and peak value (**Table 3**) of glucose infusion rate and the area under the GIR curve of plasma insulin in each part (**Table 4**). During the test, the infusion rate of 20% glucose solution was adjusted according to the blood glucose test results to maintain the target blood glucose level. **Figure 2** shows that the serum glucose concentrations at the three different injection sites were basically the same as the target blood glucose level and the blood glucose level within 3 hours was basically maintained at the target blood glucose level. The glucose infusion rate reflects the use of glucose by the tissue and its sensitivity to insulin. With the hyperinsulin-normal blood glucose clamp model in this study, GIR reflects the sensitivity of the tissue to exogenous insulin. It can be seen from **Figure 3** that glucose infusion rate curves after injections at three different positions in the abdomen, upper arm, and thigh were basically consistent with each other, which showed that exogenous insulin sensitivity was not affected by different injection sites. We also analyzed the correlation between the injection site thickness and the time to peak and rate of plasma insulin, and the area under the GIR curve, and did not find a statistically significant correlation, indicating that insulin absorption was similar at different injection sites, which was independent of the skin thickness at the injection sites. Studies on routine insulin injections have found that the thickness of injection sites and local blood flow both affect insulin absorption (22). For the first time, we conducted a study to compare the absorption of needle-free insulin injected in different parts of the body. Furthermore, we found that the absorption of insulin was not related to the skin thickness at the injection site, indicating that the absorption of the drug by jet injection (needleless) was different from that by conventional needle injection. The exact mechanism needs further study.

That being said, our study has several major limitations, and therefore, our findings should be carefully interpreted. First, the small sample size of our population made it difficult to reach statistically significant differences among the different injection sites in terms of insulin absorption. Second, subjects were followed-up for a short period of 1 week, and we are not confident whether there would be a significant variation in the absorption rate over a longer period. Third, our population was made up of normal, healthy individuals, who were administered a single dose of insulin. Therefore, future studies of larger sample sizes, longer follow-up periods, and conducted among diabetic patients who are injected with insulin regularly are still warranted to validate our findings and estimate the difference in the absorption of rapid-acting insulin through jet injectors in different body parts of diabetic patients. Finally, another point that should be carefully taken into consideration is the BMI of recruited subjects because it is evident that the absorption of insulin, regardless of body part, is variable based on BMI (23). In our study, recruited subjects had low BMI, so we are not confident whether or not our findings would be consistent among subjects with high BMI. Therefore, we recommend putting this point into consideration when conducting future studies.

The results in this study showed that the injection of insulin aspart with a needleless syringe into the abdomen, upper arm and thigh of healthy male adult volunteers had no effect on insulin absorption, and the skin thickness at the injection site had no correlation with insulin absorption. This study provided some evidence that diabetic patients took turns in needle-free insulin injections at different parts of the body, and therefore, it can be used as hypothesis-generating article for directing future research.

## Data Availability Statement

The original contributions presented in the study are included in the article/supplementary material. Further inquiries can be directed to the corresponding author.

## Ethics Statement

The studies involving human participants were reviewed and approved by the Beijing Hospital. The patients/participants provided their written informed consent to participate in this study.

## Author Contributions

QP and LG designed the manuscript. LZ, AG, and DY collected the data. XW, YZ, and LG analyzed the data. All authors listed have made a substantial, direct, and intellectual contribution to the work and approved it for publication.

## Funding

This work was supported by the National key research and development program (2020YFC2009000).

## Conflict of Interest

The authors declare that the research was conducted in the absence of any commercial or financial relationships that could be construed as a potential conflict of interest.

## Publisher’s Note

All claims expressed in this article are solely those of the authors and do not necessarily represent those of their affiliated organizations, or those of the publisher, the editors and the reviewers. Any product that may be evaluated in this article, or claim that may be made by its manufacturer, is not guaranteed or endorsed by the publisher.
